# Examining spatial variations in the relationship between domestic energy consumption and its driving factors using multiscale geographically weighted regression: a case study in Nottingham, England

**DOI:** 10.1186/s13705-025-00523-1

**Published:** 2025-05-11

**Authors:** Yuan Feng, Ying Miao, Ed Turner

**Affiliations:** Department of Society and Politics, School of Law and Social Sciences, College of Business and Social Sciences, Aston University, Birmingham, B4 7ET UK

**Keywords:** Domestic energy consumption, Geographically weighted regression, Multiscale geographically weighted regression, Lower layer super output areas

## Abstract

**Background:**

Domestic energy consumption contributes to over a quarter of the UK’s carbon emissions, understanding how it is driven can be helpful for delivering a fair energy transition to net zero. Energy usage is noted as a spatial phenomenon, however, the spatial variability of how it is driven is rarely considered in existing UK studies. To contribute to this research gap, this study examines the spatial variations in the relationship between domestic energy consumption and its driving factors using the local spatial statistical modelling technique *multiscale geographically weighted regression* (MGWR). With explanatory variables on dwelling and household characteristics, this study analyses data at Lower Layer Super Output Area (LSOA) level on the study area, Nottingham, a somewhat socio-economically deprived city that also has the UK’s largest district heating (DH) system supplying low-carbon residential heating.

**Results:**

The study reveals domestic energy consumption is driven by factors at different spatial scales with spatially varied or even spatially heterogeneous patterns. Specifically, higher domestic energy consumption is affected differently across local areas by larger percentages of dwellings with 4 or more bedrooms, unemployment, terraced dwellings, whilst by smaller percentages of social-rented housing tenures and central heating type of district heating. The impacts of dwelling energy efficiency, median household income, percentage of households with 3 or more people, fuel poverty, and central heating with renewable energy, vary across different local areas. Therefore, while there are identifiable relationships between these factors and domestic energy consumption, they differ by locality, and aggregated level analysis may fail to accurately to capture these patterns.

**Conclusions:**

Nuanced local patterns of how domestic energy consumption is driven suggest placed-based approaches and more local deliberation to devise policies may be more suitable than “one-size-fit-all” policy plans to achieve the envisioned outcomes of rapid and fair domestic energy decarbonisation and just energy transition to net zero.

## Background

Domestic energy consumption, or energy consumption of the residential sector, makes up a significant share of total energy consumption globally. Over a quarter of the UK’s total energy consumption is from domestic energy consumption, of which nearly 90% are from gas and electricity, with 80.3% used for space heating, hot water heating, and cooking, and the remaining 19.7% for lighting or appliances in 2022 [1]. While around 20% of the UK’s primary energy is from low-carbon sources, fossil fuel generated energy remains a large proportion in total energy usage [1]. With residential energy consumption constituting around a fourth of the UK’s total carbon emissions [2], domestic energy decarbonisation continues to be a major challenge in dealing with climate change [3]. In this context, developing better understanding of domestic energy consumption can be helpful for devising policies for energy decarbonisation and net zero transition [4, 5]. 

### Driving factors of domestic energy consumption in the UK

Though domestic energy consumption is traditionally studied from individual disciplinary perspectives, for instance with a specific focus on energy technologies or costs, this could overlook important contextual factors such as interactions between residents and technologies [6, 7]. Domestic energy consumption is thus widely accepted as a complex multidisciplinary phenomenon, which needs to be studied from both engineering and social science perspectives [8]. In this context, uncertainties are crucial for understanding it and devising plans for its decarbonisation [9, 10]. In reviewing literature on drivers of domestic energy consumption, this study thus adapts the taxonomy of key uncertainties from Eyre and Baruah [9] as theoretical framework. This classifies drivers into two categories by key uncertainties they contribute to: households’ demographic and economic characteristics that contribute to socio-economic uncertainties, and dwelling’s physical characteristics that contribute to socio-technical uncertainties. 

Current literature examining drivers of domestic energy consumption in the UK commonly look at a combination of explanatory variables on physical characteristics of dwellings, and the demographic, socio-economic, and sometimes the behavioural characteristics of households, with data analysed at household level [11–13], dwelling level [14], and geographical area level [4, 15, 16]. A variety of research methods have been utilised to study how these factors drive domestic energy consumption, including central tendency analysis and cross-tabulation analysis [11, 14], correlation analysis and linear regression models [4, 12, 13, 15], and data-driven models using multilayer neural network, random forest, gradient boosting algorithms [16]. A combination of dwelling and household characteristics are commonly found to be significant driving factors: dwelling types [4, 13–16], number of bedrooms [4, 11, 12, 16], energy efficiency measures [13, 14], household size or composition [4, 12, 14], income [4, 12, 14, 16], employment status [4, 12, 15], and housing tenures [4, 14]. 

#### Dwelling characteristics

Existing studies find similar results on how dwelling characteristics affect domestic energy consumption. On number of bedrooms, Gassar et al. [16] find number of rooms are strongly related to more gas consumption in London. Fuerst et al. [12] similarly find greater number of beds is associated with higher gas consumption for space heating per capita across England. On dwelling types, Huebner et al. [13] find they are significant predictors, with flat and mid-terrace significantly associated with less consumption. Wyatt [14] finds detached and flats have the highest electricity consumption, whereas detached and semi-detached have the highest gas consumption. Cheng and Steemers [15] find detached dwellings are associated with the highest consumption, whereas flats are the lowest, with semi-detached and mid- or end-terrace are with lower consumption in descending order. On dwelling energy efficiency, previous studies identify better efficiency leads to less consumption. The Domestic National Energy Efficiency Data-Framework (NEED) developed by UK Government [17] finds lower gas consumption is associated with better dwelling energy efficiency [18]. Cheng and Steemers [15] find lower boiler efficiency is associated with higher consumption; while Wyatt [14] finds energy efficiency measures, such as cavity wall insulation and loft insulation, lead to less overall consumption for all dwelling types. 

#### Household characteristics

Several household characteristics are commonly identified as important explanatory factors [19]. Income is a significant factor widely discovered to be positively associated with domestic energy consumption in existing UK literature [4, 12, 15, 16]. Gassar et al. [16] find higher household income is associated with higher consumption and identified income as the most influential factor. Fuerst et al. [12] find income to be more important than dwelling characteristics in explaining per capita energy consumption. Wyatt [14] finds higher income is consistently associated with greater residential gas and electricity consumption. On employment, Cheng and Steemers [15] find better employment status, which also manifest via type and size of dwellings households occupy, is correlated with higher consumption. On social housing, Wyatt [14] finds it is associated with less gas and electricity consumption. While for household size, Druckman and Jackson [4], Wyatt [14], Huebner et al. [13], and Domestic NEED [18] find higher consumption is related to larger household size or greater number of adult residents in household, whereas Fuerst et al. [12] find it is negatively correlated with per capita gas consumption.

#### Knowledge gaps

Although the literature has provided valuable insights into this research topic, there are still considerable knowledge gaps that require further investigation. The comprehensive review by Frederiks et al. [19] finds in existing research, while socio-demographic factors are widely identified as important for explaining domestic energy consumption, how they influence its variability remains inconsistent and inconclusive. For levels at which domestic energy consumption are studied, the critical review of Harputlugil and de Wilde [20] points out much current research mostly focuses on a small number of buildings and individuals. Baker and Rylatt [11] also mention small-scale studies that meticulously monitor small numbers of dwellings, which had been the case for many UK studies, could lead to the problem of not being applicable to wider areas. Energy poverty, referred to as fuel poverty in the UK [21], is a situation of lack of access to adequate energy [21–23]. In a review by Han and Wei [24] on household energy consumption studies worldwide, energy poverty is identified as an emerging research frontier in domestic energy consumption research. Meanwhile, Langevin et al. [25] note low-income housing is an important aspect often overlooked in domestic energy consumption and its reduction, though this is rarely investigated in existing UK studies. Moreover, the review of UK’s bottom-up building stock models of residential energy consumption by Kavgic et al. [26] identifies a major issue on uncertainties in socio-technical drivers of domestic energy consumption. Harputlugil and de Wilde [20] also highlight this issue, such as how consumption is affected by households using different types of central heating or behavioural changes from energy efficiency measures, and call for this knowledge gap to be addressed in future research.

Furthermore, domestic energy consumption is widely noted as a spatial phenomenon in research worldwide [27, 28], from the USA [29–31], China [32, 33], to European countries [34–36], including the UK [37–41]. However, for UK research, Cheng and Steemers [15] note a limitation in previous domestic energy consumption models is that their spatial resolution is predominantly at the aggregated national level, which could become inaccurate at the more disaggregated local levels, such as region and local authority. They hence encourage sub-national level models that can allow more detailed study at finer spatial resolution, which can be helpful for informing local governments’ energy-related policymaking, such as identifying target areas for energy efficiency interventions. On the other hand, Love and Cooper [42] note the lack of spatial properties in research design, which prompt further consideration on spatial effects in studying the relationship between domestic energy consumption and its driving factors. Additionally, to understand local dynamics to inform regional policies, it is important to recognise spatial heterogeneity in local characteristics and how they affect the target variables [43]. This aspect, however, is often overlooked with global measures used in traditional linear regression models [44]. This issue is also found to be the case in the aforementioned literature, where global regression models such as multiple linear regression (MLR) models are commonly applied [4, 12, 13, 15].

### Exploring spatial variability in how domestic energy consumption is driven

Against this backdrop, this study innovatively applies the local spatial statistical modelling technique, geographically weighted regression (GWR), to examine the spatial variations in the relationship between domestic energy consumption and its driving factors. Similar to Tobler’s first law of geography which states “everything is related to everything else, but near things are more related than distant things” [45], GWR builds on the assumption that nearby observations have more influence on parameter estimates than observations that are more distant [46]. GWR is designed to deal with and explore spatial heterogeneity or non-stationarity in regression relationships, where relationships between variables cannot be simply explained by a global model [47–51]. GWR allows relationships in a regression model to vary over space, hence enables the exploration of spatial variability in the relationship between the dependent variable and independent variables [52–54]. Moreover, local results from GWR can also be visualised with mapping for presenting their policy implications to stakeholders [55–57]. These features therefore make GWR a suitable technique to study the spatial variability in the relationship between domestic energy consumption and its driving factors in the UK, with potential to address the knowledge gap on spatial properties and inform regional policymaking with interpretable maps visualising local relationships.

In literature worldwide, GWR has been applied in environmental and socio-economic research [58] as well as interdisciplinary topics [59]. In UK literature, GWR has also been used to examine a wide range of research topics, from air pollution [60], land value [61, 62], environmental conservation [63–65], to education performance [66, 67], public health [68–70], domestic violence [71, 72], overcrowding [73], migration [74], fuel poverty [75], and low-carbon energy technology uptake [76]. In energy research worldwide, GWR has been applied in topics ranging from CO2 emissions from energy consumption [77–79], energy prices [80], energy expenditures [81], to energy justice [82]. However, for research on domestic energy consumption, GWR is only found to be applied on two other countries, respectively, on factors for electricity consumption in Korea [83] and determinants of household energy consumption in the Netherlands [84, 85].

To contribute to the above-mentioned knowledge gaps and the emerging literature using GWR to explore domestic energy consumption, this study aims to examine the spatial variability in the relationship between domestic energy consumption and its driving factors. Based on the reviewed literature, it explanatory variables related to household and dwelling characteristics that are commonly found to be influential, as well as emerging variables that warrant further research. For dwelling characteristics, the study includes the number of bedrooms, dwelling types, energy efficiency, and central heating types. For household characteristics, it includes household size, income, employment status, fuel poverty, and housing tenure.

This study focuses on the unique study area Nottingham, a somewhat socio-economically deprived English city [86] which also has the UK’s largest district heating (DH) network supplying low-carbon residential heating [87]. Analysis is conducted at the spatial level of Lower Layer Super Output Area (LSOA). LSOAs are geographical areas for census statistics, they are made up of groups of 4 or 5 Output Areas (OAs) which are the lowest level of geographical area. Each LSOA comprises from 400 to 1200 households, or a residential population between 1000 and 3000 [88]. This level of data analysis and study area is chosen to address the research gap that previous literature either focused narrowly on certain buildings, missing boarder trends [11, 20], or was too generalised at the national level to reveal robust local dynamics [15].

Firstly, a MLR model is developed and Moran's $I$ and Geary’s $C$ are tested on the residuals to check for spatial autocorrelation [89]. A spatial error model (SEM) is then estimated, which incorporates spatial autocorrelation by including a spatial autoregressive error term [90], to validate the advantages of GWR in investigating relationship with spatial autocorrelation. Next, a GWR model is developed to examine the spatial variability in the relationship between domestic energy consumption and explanatory variables across Nottingham LSOAs. To explore how the spatial variability can be best explained, a multiscale geographically weighted regression (MGWR) model is further developed to investigate the different spatial scales in this relationship.

## Methods

### Study area

Nottingham is a city in East Midlands, England, with 124,740 households and 134,402 dwellings [91, 92]. Nottingham has the largest district heating network in the UK [87]. Powered by municipal waste incineration, the Nottingham district heating network has been providing residential energy for space heating and hot water to over 5000 dwellings for three decades [93]. However, fuel poverty and deprivation are both severe in Nottingham. In 2021, 18.3% of Nottingham households are in fuel poverty, much higher than East Midlands’ regional average of 13.6%, and the England average of 13.1% [94, 95]. The Index of Multiple Deprivation (IMD), which measures the relative general socio-economic deprivation, ranks Nottingham the 11th most deprived among 317 local authorities across England [96]. Within Nottingham, local deprivation situations vary widely. While 4.4% of Nottingham LSOAs are in the 20% least deprived in England, 30.8% fall amongst the 10% most deprived, and 57.1% fall in the 20% most deprived [96].

### Data collection

This study uses public datasets from Census 2021 and Office for National Statistics (ONS) for the dependent variable domestic energy consumption and explanatory variables on dwelling and household characteristics. Their information and data sources are presented in Table 1.

**Table 1:** Variable information and data sources

Variable	Unit	Data description	Data level	Year	Data sources
Domestic.energy.consumption	kWh	Sum of annualised total domestic gas consumption and annualised total domestic electricity consumption	LSOA	2021	Lower and Middle Super Output Areas gas consumption [97] Lower and Middle Super Output Areas electricity consumption [98]
Bedrooms.4.or.more	%	Percentage of dwellings with number of bedrooms being 4 or more	LSOA	2021	Number of bedrooms [99]
Dwelling.terraced	%	Percentage of terraced dwellings	LSOA	2021	Accommodation type [100]
Energy.efficiency.score	number	Median Energy Efficiency Score of all dwellings, measured by Energy Efficiency Certificate (EPC)	MSOA, converted to LSOA	2021	Energy efficiency of Housing [101]
Heating.renewable.energy	%	Central heating type being renewable energy, categorised as "renewable energy only"	LSOA	2021	Type of central heating in household [102]
Heating.district.heating	%	Central heating type being district heating, categorised as "district or communal heat networks only"	LSOA	2021	Type of central heating in household [102]
Household.3.or.more.people	%	Percentage of households with household size being three or more people	LSOA	2021	Household size [103]
Income	£	Mean net annual household disposable income (equivalised) before housing costs	MSOA, converted to LSOA	2020	Income estimates for small areas [104]
Unemployed	%	Percentage of the unemployed, categorised as “never worked and long-term unemployed”	LSOA	2021	National Statistics Socio-economic Classification (NS-SEC) [105]
Fuel.poverty	%	Percentage of households in fuel poverty, measured by the Low Income Low Energy Efficiency (LILEE) indicator	LSOA	2021	Fuel poverty statistics [94]
Social.rented	%	Percentage of housing tenure type being social-rented	LSOA	2021	Tenure [106]

#### Dependent variable

**Fig. 1:**
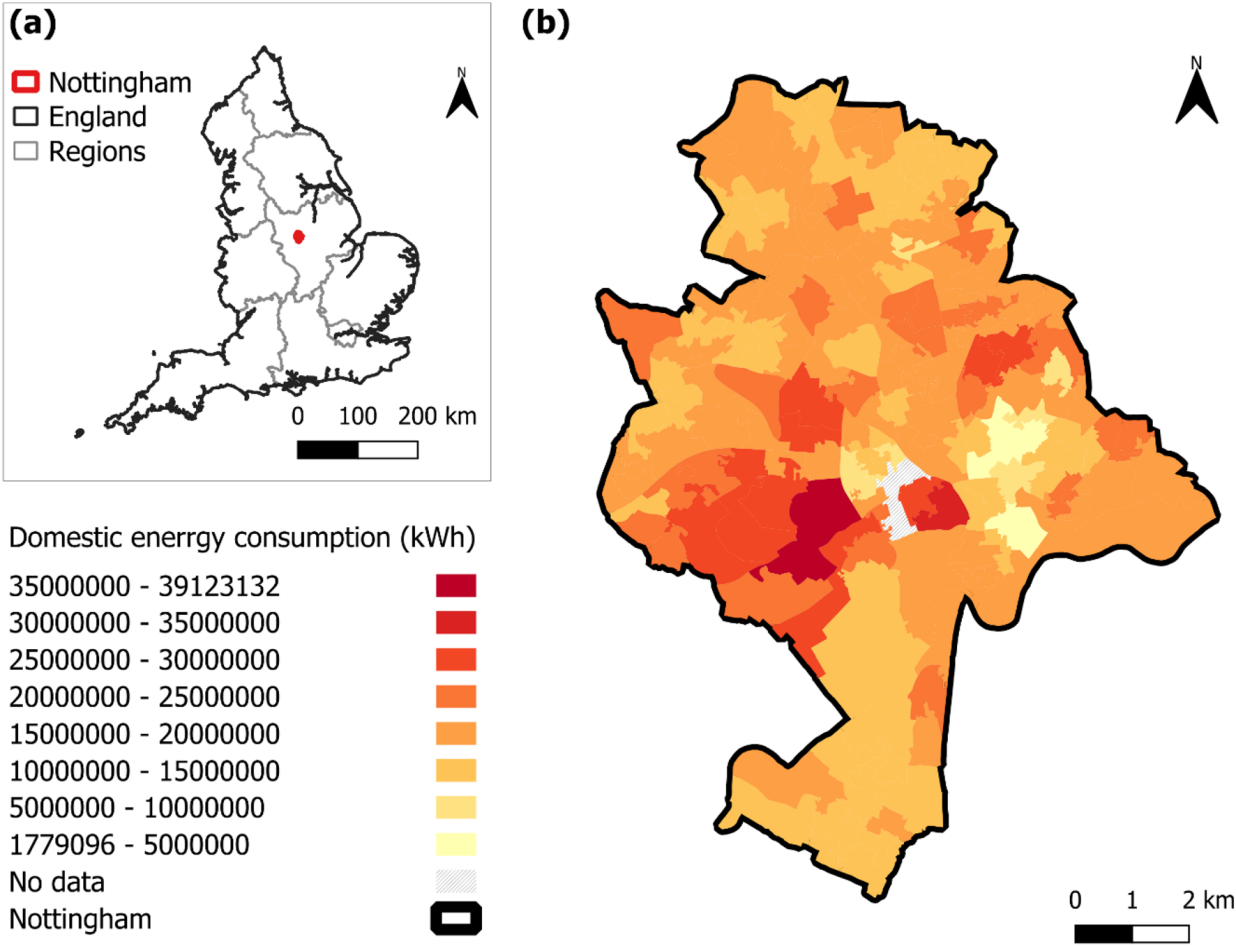
Study area and dependent variable: **a** Nottingham in East Midlands, England; **b** domestic energy consumption across Nottingham LSOAs

Domestic energy consumption is calculated as the sum of annualised total domestic gas consumption [97] and annualised total domestic electricity consumption [98] at LSOA level. These two GOV.UK datasets use Census 2011 LSOA boundaries, as most datasets for explanatory variables use Census 2021 LSOA boundaries, the latter is used for analysis. In Nottingham, from 2011 to 2021 boundaries, 10 smaller previous LSOAs merged into 5 current larger LSOAs, for their total domestic energy consumption, the sum of their previous components is calculated; for the 4 smaller current LSOAs divided from 2 previous larger ones, their domestic energy consumption data are not available, so they are excluded from the analysis. Figure 1 maps domestic energy consumption across the 175 Nottingham LSOAs, ranging from 1,779,096 to 39,123,132 with a mean of 16,504,060 kWh.

#### Explanatory variables

*Number of bedrooms.* Dwellings are self-contained units of household accommodation, 3-bedroom dwellings are most common across England (40%), followed by 2-bedroom (27.3%), 4-or-more-bedrooms (21.1%), and 1-bedroom (11.6%) [107, 108]. To look at how dwellings with more bedrooms than usual affect domestic energy consumption, the percentage of dwellings with 4 or more bedrooms is included [99]. This ranges from 1.1 to 28.8% across Nottingham LSOAs, with a mean of 6.3%, which is lower than the overall level of 21.1% in England.

*Dwelling types.* In England, terraced dwellings are the second most common (23.0%) after semi-detached (31.5%), followed by detached (22.9%) and flat (17.1%) [107, 108]. To look at how this common dwelling type sharing multiple walls with neighbours affect domestic energy consumption, the percentage of terraced dwellings is included [100]. This ranges from 0.41% to 69.1% across Nottingham LSOAs, with a mean of 27.6%, which is higher than the overall level of 23.0% in England.

*Energy efficiency of housing.* Energy Performance Certificates (EPC) assess intrinsic energy efficiency based on a dwelling’s physical characteristics, its ratings from A to G represent energy efficiency scores between 100 and 0, with A (over 92) the most energy efficient, B (81–91), C (69–80), D (55–68), E (39–54), F (21–38) and G (1–20) the least [109]. This dataset is aggregated EPC energy efficiency scores in 2021, though only published at Middle Layer Super Output Area (MSOA) level [101]. MSOAs are the upper-level geographical area of LSOAs, each MSOA contains 4 or 5 LSOAs [88]. To look at how varied levels of dwellings’ energy efficiency affect domestic energy consumption, median energy efficiency scores are included. To be consistent with the LSOA level of analysis, MSOA level data are converted to LSOA level, via LSOA-to-MSOA lookup that assigns data on median energy score from MSOAs to the LSOAs they contain [110]. Median energy efficiency score ranges from 58 to 77 across Nottingham LSOAs, with a mean of 66.1, which is slightly higher than the overall level (66, D equivalent) in England [109].

*Central heating types.* The most common type of central heating in England is “main gas only” (74.0%, 17.3 million households), followed by “electric only” (8.7%, 2.0 million), “two or more types of central heating (not including renewable energy)” (8.5%, 2.0 million); while “district or communal heat networks only” and “renewable energy only”, respectively, takes up 0.9% (217,000 households) and 0.4% (92,000 households) [111]. Considering the widespread residential coverage of Nottingham’s district heating network and Nottingham City Council’s renewable energy initiatives [112], to look at how these two central heating types affect domestic energy consumption, the percentages of renewable energy and district heating are included, respectively [102]. Across Nottingham LSOA, renewable energy and district heating, respectively, ranges from 0 to 2.6% with a mean of 0.66%, and from 0 to 49.1% with a mean of 2.5%, both are much higher than the overall levels in England.

*Household size.* In England, the most common size of household is 2 people, followed by 1, 3, and 4 or more people, with the average being 2.4 people [113]. To look at how households with higher-than-average size affect domestic energy consumption, the percentage of households with 3 or more people is included [103]. This ranges from 4.7% to 60.1% with a mean of 36.5% across Nottingham LSOAs.

*Household income.* For the mean net annual household disposable income, equivalised for household size to account for achieving the same living standard, across MSOAs in England and Wales, 2 of Nottingham MSOAs are among the bottom 50 [114]. The data on the mean net annual household disposable income (equivalised) before housing costs (BHC) are on the financial year ending (FYE) 2020, though only published at MSOA level [104]. To look at how varied levels of income affect domestic energy consumption, mean household income is included. To maintain consistency with the LSOA level of analysis, this MSOA level data is disaggregated to the LSOA level using an LSOA-to-MSOA lookup, which assigns mean household income data from MSOAs to the LSOAs they contain [110]. Household income ranges from £18,800 to £46,100 across Nottingham LSOAs, with a mean of £26,078.9. This mean falls within the lowest 25th percentile (£28,500) in England and Wales.

*Employment status.* National Statistics Socio-economic Classification (NS-SEC) indicates a person's socio-economic position based on their occupation and other job characteristics. Across England and Wales, the “never worked and long-term unemployed” makes up 8.5% or 4.1 million [115]. From the NS-SEC data [105], to look at how unemployment affects domestic energy consumption, the percentage of the unemployed is included. This ranges from 1.08% to 18.24%, with a mean of 9.13% across Nottingham LSOAs, which is higher than the overall unemployment level in England and Wales.

*Fuel poverty.* Low Income Low Energy Efficiency (LILEE) is the current fuel poverty indicator in England, it estimates fuel poverty based on the relationship between three main drivers: “energy efficiency of the home”, “household income”, “energy prices” [116]. Fuel poverty is measured as a collective outcome of low housing energy efficiency, low household income, and high energy prices. Specifically, LILEE determines a household to be fuel poor with two conditions: living in a dwelling with EPC rating below C, and with disposable income after housing costs (AHC) and energy needs being below poverty line. From this dataset [94], to look at how varied levels of fuel poverty affect domestic energy consumption, the percentage of fuel poor households is included. This ranges from 6.5 to 41.6% across Nottingham LSOAs with a mean of 17.9%, which is higher than 13.1%, the overall level of fuel poverty in England [95].

*Housing tenures.* In England, the most common major types of housing tenures are, in descending order, owner-occupied (62.4%), private-rented (20.4%), and social-rented (through local councils or housing associations) (17.1%) [107]. To look at the emerging though less studied topic of how housing principally accessible to those on lower incomes affects domestic energy consumption, the percentage of social-rented is included [106]. This ranges from 0.8 to 82.1% across Nottingham LSOAs, with a mean of 26.1%, which is higher than the overall 17.1% in England. Table 2 summarises descriptive data of all variables.

**Table 2:** Descriptive statistics of variables

Category	Variable	Mean	Min	Max	SD
	Domestic.energy.consumption	16504060.02	1779096.00	39123132.00	5301560.92
Dwelling characteristics	Bedrooms.4.or.more	6.34	1.10	28.77	5.27
	Dwelling.terraced	27.58	0.41	69.10	16.88
	Energy.efficiency.score	66.06	58.00	77.00	4.51
	Heating.renewable.energy	0.66	0.00	2.60	0.52
	Heating.district.heating	2.51	0.00	49.06	7.46
Household characteristics	Household.3.or.more.people	36.48	4.70	60.10	9.80
	Income	26078.86	18800.00	46100.00	5218.32
	Unemployed	9.13	1.08	18.24	3.24
	Fuel.poverty	17.86	6.50	41.60	8.00
	Social.rented	26.06	0.80	82.10	17.39

### Multiple linear regression

MLR using ordinary least squares (OLS) calculates the relationship between the dependent variable and independent variables, allowing the examination of specific effects between them [117, 118], with key assumptions that observations across study areas are independent with constant variance, and error terms are not correlated, its equation is shown in Eq. (1) [119, 120]:

1 $$Y_{i} = \beta_{0} + \mathop \sum \limits_{k = 1}^{n} \beta_{k} x_{ik} + \varepsilon_{i} ,$$

where ${Y}_{i}$ is the dependent variable, ${\beta }_{0}$ is the intercept, ${x}_{ik}$ is the $k$ th independent variable at the point $i$, ${\beta }_{k}$ is the regression coefficient of the $k$ th independent variable, and ${\varepsilon }_{i}$ is the random error term. Using domestic energy consumption as the dependent variable and various explanatory variables, an MLR model is first developed to check for spatial correlation in its residuals. If spatial correlation is present, it would violate the MLR model's key assumption regarding error terms, making it unsuitable for investigating the relationship between domestic energy consumption and the explanatory variables.

### Tests of spatial autocorrelation

To check for spatial correlation in the MLR model’s residuals, Moran's $I$ and Geary’s $C$, the two most common indices for assessing spatial autocorrelation [89, 121], are used. Moran's $I$ measures how correlated are the values of neighbouring spatial objects, which indicates patterns of spatial clustering [76] and is commonly used for detecting whether spatial autocorrelation effects are present [122]. Its values ranges from − 1 to 1, with positive values suggesting tendency for clustering whereas negative for dispersion, its formula is shown in Eq. (2) [123]:

2 $$Moran^{\prime}s I = \frac{n}{{\mathop \sum \nolimits_{i = 1 }^{n} \mathop \sum \nolimits_{j = 1}^{n} w_{ij} }}\, \cdot \,\frac{{\mathop \sum \nolimits_{i = 1 }^{n} \mathop \sum \nolimits_{j = 1}^{n} w_{ij} \left( {x_{i} - \overline{x}} \right)\left( {x_{j} - \overline{x}} \right)}}{{\mathop \sum \nolimits_{i = 1}^{n} \left( {x_{i} - \overline{x}} \right)^{2} }},$$

where $n$ is the total number of spatial objects in the study area, in this case LSOAs in Nottingham, ${w}_{ij}$ is an element of the inverse distance spatial weight matrix $w$, which indicates the neighbouring relationship of spatial units of $n$ locations; ${x}_{i}$ and ${x}_{j}$ are values of the variable $x$ at the coordinates $i$ and $j$, $\overline{x }$ is the average of all observations for the variable $x$ of the $n$ spatial objects.

Geary’s $C$ is more sensitive to difference in values between pairs of compared observations than the co-variation between the pairs, thus indicating more local variations compared to the more global indicator of Moran's $I$ [124]. Geary’s $C$ ranges from 0 to positive values, with value of 1 indicates no spatial autocorrelation, values below 1 representing increasing positive spatial autocorrelation, and above 1 indicating increasing negative spatial autocorrelation, its formula is shown in Eq. (3) [89]:

3 $$Geary^{\prime}s C = \frac{n - 1}{{2\mathop \sum \nolimits_{i = 1 }^{n} \mathop \sum \nolimits_{j = 1}^{n} w_{ij} }}\, \cdot \,\frac{{\mathop \sum \nolimits_{i = 1 }^{n} \mathop \sum \nolimits_{j = 1}^{n} w_{ij} \left( {x_{i} - x_{j} } \right)^{2} }}{{\mathop \sum \nolimits_{i = 1}^{n} \left( {x_{i} - \overline{x}} \right)^{2} }},$$

where ${w}_{ij}$, $n$, ${x}_{i}$, ${x}_{j}$, $\overline{x }$ are analogous to those in Moran's $I$. As Nottingham LSOAs are neighbouring polygons sharing boundaries, for both indicators, the spatial weight matrix is created using the first-order Queens’ contiguity approach.

### Spatial error model

To validate the advantages of GWR in investigating relationships with spatial autocorrelation, a SEM incorporating a spatial autoregressive error term [90] is developed before proceeding to GWR. SEM achieves this by assuming spatial dependence in OLS residuals, it decomposes OLS residuals into two components: the spatially dependent error and the spatially independent random error, its formula is shown in Eq. (4) [125, 126]:

4 $$Y_{i} = \mathop \sum \limits_{k = 1}^{n} \beta_{k} x_{ik} + \lambda w_{i} \mu_{i} + \varepsilon_{i} ,$$

where ${Y}_{i}$, ${x}_{ik}$, ${\beta }_{k}$ are analogous to those in OLS, ${\mu }_{i}$ is the spatially dependent error at location $i$; ${w}_{i}$ is the spatial weight matrix; $\lambda$ is the coefficient of the spatially dependent errors; and ${\varepsilon }_{i}$ is the spatially independent random error. For the spatial error coefficient $\lambda$, a positive value indicates positive spatial dependence, whereas negative value indicates negative spatial dependence; a statistically significant $\lambda$ suggests spatial dependence matters in explaining variations in the dependent variable [127], which can help validate advantages of GWR in investigating the relationship between domestic energy consumption and the explanatory variables.

### Geographically weighted regression

In comparison to the global MLR model that uses constant regression coefficients to explain the whole study area, by accounting for spatial structure, GWR extends MLR to produce a separate model and local parameter estimates for each location [128, 129]. To examine the spatially varying relationship between domestic energy consumption and explanatory variables across Nottingham LSOAs, a GWR model is developed. GWR allows its model coefficients to vary spatially, it carries out location-based calibration of linear regression by giving greater weights on observations that are nearer to each regression point, its equation is shown in Eq. (5) [50, 130, 131]:

5 $$Y_{i} = \beta_{0} \left( {u_{i} ,v_{i} } \right) + \mathop \sum \limits_{k = 1}^{n} \beta_{k} \left( {u_{i} ,v_{i} } \right)x_{ik} + \varepsilon_{i} ,$$

where ${Y}_{i}$ is the dependent variable at location $i$, $\left({u}_{i},{v}_{i}\right)$ is the spatial coordinates of location $i$, ${x}_{ik}$ is the $k$ th independent variable at location $i$, ${\beta }_{k}\left({u}_{i},{v}_{i}\right)$ is the local regression coefficient for the $k$ th independent variable at location $i$, and ${\varepsilon }_{i}$ is the random error term at location $i$. GWR uses one single bandwidth for all variables [55]. In GWR, alternative to fixed bandwidth of one single geometric distance with varied number of neighbours, adaptive bandwidth uses constant number of neighbours and varied distance for each local observation, whose weight and coefficient are determined by the optimal distance between its neighbours and its centre [132]. GWR with adaptive bandwidth can thus consider how the relationship between variables vary spatially while reducing Modifiable Area Unit Problem (MAUP), the problem of inconsistent results created by artificially defined spatial boundaries, such as LSOAs, and improving fitting result of GWR [133, 134]. Adaptive bandwidth is hence applied in GWR.

### Multiscale geographically weighted regression

Extending from GWR which applies one single bandwidth to all variables, MGWR allows relationships between dependent variable and explanatory variables to vary individually at different bandwidths or spatial scales [55]. Considering the explanatory variables representing varied local characteristics may interact with domestic energy consumption at different spatial scales, an MGWR model is further developed. For each explanatory variable, MGWR separately determines an optimal bandwidth based on the best Akaike Information Criterion (AIC) and its corrected version (AICc) [58, 135]. MGWR is thus more flexible in considering the complexities in the spatially varying relationships, and can provide important information on the varied spatial scales at different explanatory variables interact with the dependent variable, its equation is shown in Eq. (6) [136, 137]:

6 $$Y_{i} = \beta_{{bw_{0} }} \left( {u_{i} ,v_{i} } \right) + \mathop \sum \limits_{k = 1}^{n} \beta_{{bw_{k} }} \left( {u_{i} ,v_{i} } \right)x_{ik} + \varepsilon_{i} ,$$

where ${Y}_{i}$ is the dependent variable at location $i$, $\left({u}_{i},{v}_{i}\right)$ is the spatial coordinates of location $i$, ${\beta }_{{bw}_{k}}\left({u}_{i},{v}_{i}\right)$ is the local regression coefficient for the $k$ th independent variable at location $i$, ${bw}_{k}$ is the optimal bandwidth assigned for the $k$ th explanatory variable, ${\varepsilon }_{i}$ is the random error term at location $i$.

In this study, the open-source software QGIS (version 3.34.2) [138] is used for geospatial data processing and mapping; the open-source software RStudio with the programming language R (version 4.3.2) [139] is used for statistical data processing and modelling.

## Results

### Model performance comparison

The results of the global MLR model are summarised in Table 3. The Variance Inflation Factor (VIF) is calculated to detect issues of multicollinearity among explanatory variables. The VIF values are between 1.359 and 2.450, which are below the common threshold of 5, suggesting low level of multicollinearity that does not bias model estimates. On residuals of MLR, Moran's $I$ is statistically significant at 0.174 and Geary’s $C$ is statistically significant at 0.876, both suggesting significant positive spatial autocorrelation is present. This indicates MLR’s error terms are spatially correlated, hence renders the global MLR model unsuitable for explaining this relationship. The SEM shows a positive $\lambda$ value statistically significant at 0.41016 (*p* = 0.00025663), which further demonstrate spatial dependence matters in explaining variations in the dependent variable, thus validating the advantages of GWR and MGWR in investigating this relationship. Therefore, the GWR and MGWR models are developed to investigate the spatially varying relationship between domestic energy consumption and the explanatory variables across Nottingham LSOAs.

**Table 3:** MLR results with significant positive spatial correlations detected in model residuals

	Coefficient	VIF	*p*-values
Household.3.or.more.people	9,311	2.280	0.818
Income	161 **	2.344	0.038
Unemployed	242,353**	2.048	0.038
Fuel.poverty	−11,730	1.954	0.798
Social.rented	−67,775***	2.166	0.003
Bedrooms.4.or.more	411,999***	2.450	0.0000004
Dwelling.terraced	12,335	1.964	0.572
Energy.efficiency.score	−246,906***	1.640	0.001
Heating.renewable.energy	113,096	1.359	0.848
Heating.district.heating	−178,944***	1.573	0.00008
Intercept	25,450,754***	–	0.00005
Observations	175		–
Moran's I of Residuals	0.174***		0.00006
Geary's C of Residuals	0.876**		0.01546

^*^p < 0.1; **p < 0.05; ***p < *0.01*

Table 4 compares the goodness-of-fit of the MLR, GWR, and MGWR models using the same explanatory variables, with diagnostic measures of AICc and residual sum of squares (RSS). The MLR yields a low coefficient of determination, an adjusted R2 of 0.573, indicating 42.7% of variations in domestic energy consumption remain unexplained. For the GWR model using adaptive bandwidth, comparing to MLR, values of RSS and AICc decreased while the adjusted R2 increased slightly to 0.589, indicating a better model fit. For the MGWR model assigning individual spatial scales for each explanatory variable, RSS and AICc further decreased to the lowest values, and the adjusted R2 increased to the highest value of 0.754, demonstrating the best model fit of all.

**Table 4:** Model fit of MLR, GWR, MGWR

	MLR	GWR	MGWR
AICc	5782	5780	5737
R2	0.597	0.647	0.847
Adjusted R2	0.573	0.589	0.754
RSS	1.97E + 15	1.73E + 15	7.51E + 14

### Spatial scales of explanatory variables in GWR and MGWR

The optimal bandwidths of explanatory variables in GWR and MGWR are compared in Table 5. GWR’s adaptive bandwidth is relatively large at 161, suggesting the spatial scale fitted to all explanatory variables covers 92% of the 175 Nottingham LSOAs. In comparison, the optimal bandwidths chosen in MGWR reveal more nuancedly varied spatial scales among the explanatory variables.

**Table 5:** Comparison of optimal bandwidths in GWR and MGWR

	Bandwidth	
	GWR	MGWR
Household.3.or.more.people	161	22
Income	161	61
Unemployed	161	165
Fuel.poverty	161	73
Social.rented	161	173
Bedrooms.4.or.more	161	173
Dwelling.terraced	161	173
Energy.efficiency.score	161	18
Heating.renewable.energy	161	173
Heating.district.heating	161	173
Intercept	161	173

For the MGWR, the smallest bandwidths are found in energy efficiency score (18, covering 10.3% Nottingham LSOAs) and household of 3 or more people (22, 12.6%), which indicates effects of these two explanatory variables on domestic energy consumption are most local at the smallest spatial scales. Income and fuel poverty are also discovered to have relatively smaller bandwidths of 61 (34.9%) and 73 (41.7%), indicating they also affect domestic energy consumption more locally at smaller spatial scales. The rest of the explanatory variables have relatively larger optimal bandwidths: for unemployment this is 165 (94.3%), and the rest is 173 (98.6%), including social rented, 4-or-more bedrooms, terraced dwellings, renewable energy, and district heating.

### Spatial variability of local relationships in GWR and MGWR

**Fig. 2:**
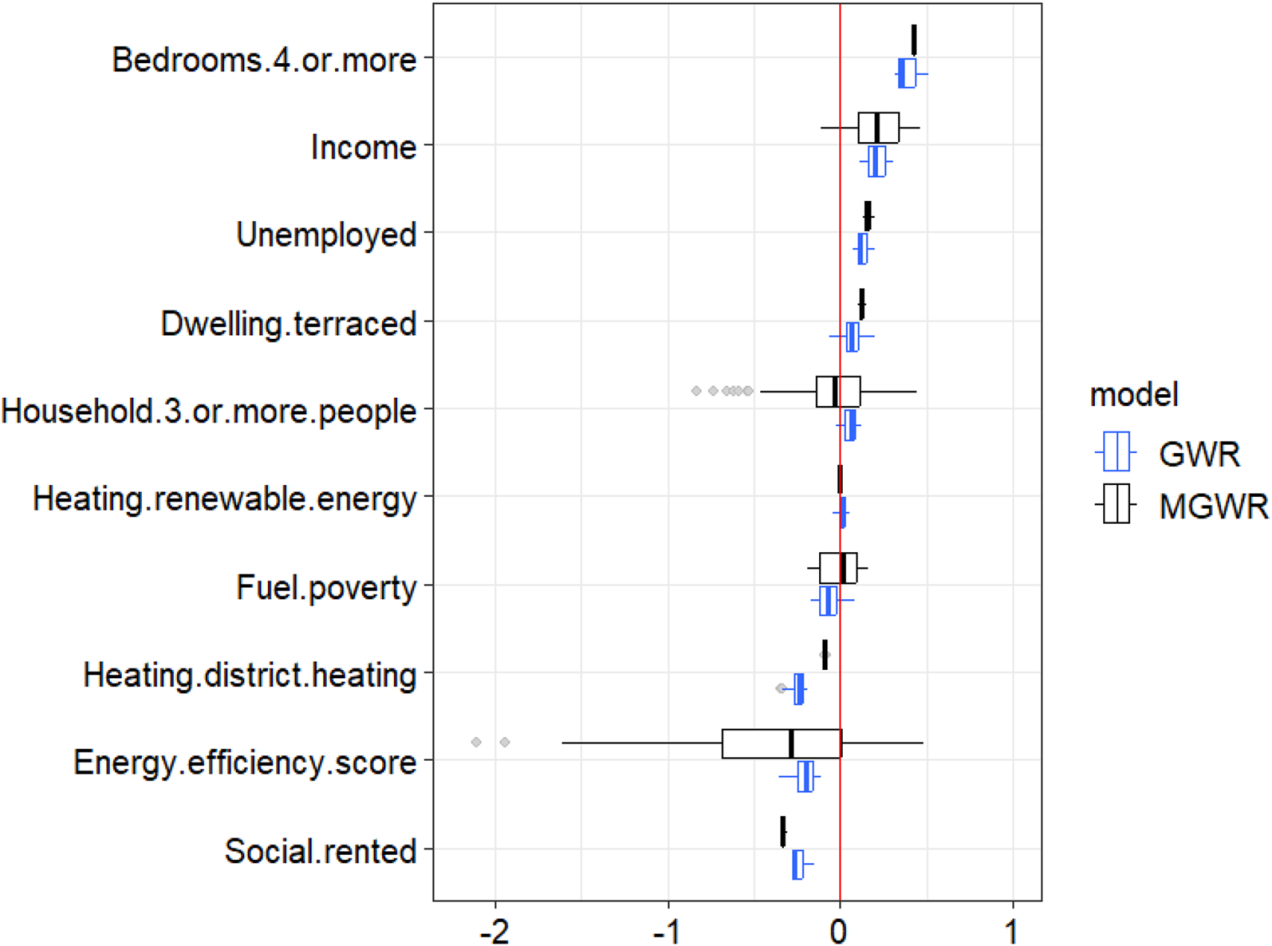
Variability in local coefficients of GWR (in blue) and MGWR (in black): explanatory variables ordered by median values of standardised local coefficients; red line denotes value 0 highlight variables with bidirectional relationships; grey dots represent outliers

**Fig. 3:**
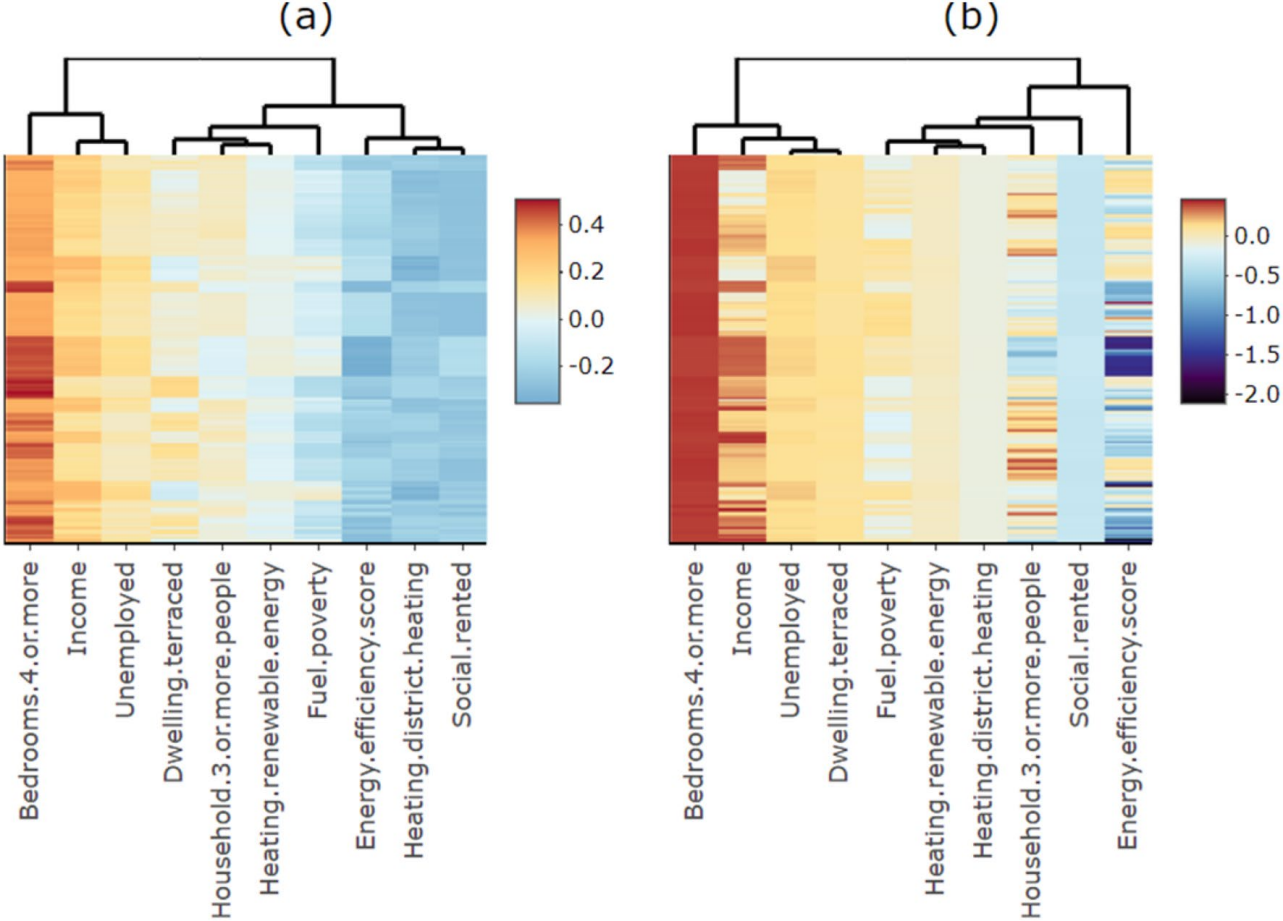
Heatmap of standardised local coefficients: comparison of effects on domestic energy consumption between a GWR and b MGWR

The local coefficients of explanatory variables in GWR and MGWR are summarised in Table 6. Figure 2 standardises data in Table 6 to show variability of local coefficients in GWR (blue) and MGWR (black). It compares the influence of explanatory variables’ effects in the two models, where larger positive values and smaller negative values are more influential. The variables are ordered by the median values of their standardised local coefficients for easier comparison, the red vertical line denoting value 0 highlights explanatory variables with bidirectional local relationship, grey dots represent outliers. Heatmaps in Fig. 3 further compare patterns of explanatory variables’ effects on domestic energy consumption in GWR (Fig. 3a) and MGWR (Fig. 3b), by visualising their standardised local coefficients in rows of Nottingham LSOAs and columns of explanatory variables. They are ordered so that values of greater similarity are near each other, to highlight patterns denoted by dendrograms of hierarchical clustering trees on heatmaps' margins [140]. This is controlled using the “optimal-leaf-order” seriation rotating the branches to minimise the sum of distances between each adjacent leaf with the R package *heatmaply* [141]. For GWR, the most influential driving factors are 4-or-more bedrooms, income, social-rented, district heating, and energy efficiency score. For MGWR, the most influential ones are found to be energy efficiency score, 4-or-more bedrooms, social-rented, income, and unemployment.

**Table 6:** Summary of model coefficients of GWR and MGWR

	GWR			MGWR		
	Min	Mean	Max	Min	Mean	Max
Household.3.or.more.people	−13846.1	30021.5	64007.7	−448579.3	−20988	238211.7
Income	116.5	210.4	313.3	−115.4	200.6	474.7
Unemployed	121366.6	203156.6	325301.7	210485.7	258386.7	331162.9
Fuel.poverty	−110787.7	−44604.5	53025	−126273.2	−3100.7	107977.5
Social.rented	−84893.5	−73969.1	−45452.1	−104358.3	−100874.9	−92324
Bedrooms.4.or.more	314349	381836.3	515053.1	418369.5	428593.6	438475.6
Dwelling.terraced	−21154	20480.3	62696.6	32353.3	38683.5	46304.4
Energy.efficiency.score	−415979.3	−250336.6	−129345.7	−2479471	−488074.4	562632.2
Heating.renewable.energy	−418733.6	96092.6	510910.6	−165766.6	−11625	169107.7
Heating.district.heating	−247768.3	−174416	−132806.1	−68662.5	−64505.4	−61549.6
Intercept	14378551.9	24839514.1	32323635.4	−30422982	42031811.6	170034083

For GWR, 3 explanatory variables are found with positive effects on domestic energy consumption varying spatially across Nottingham LSOAs: 4-or-more bedrooms, income, and unemployment; meanwhile, 3 are found with spatially varied negative effects: district heating, social-rented, and energy efficiency scores. For explanatory variables with spatially heterogeneous effects, 4 are discovered in GWR: fuel poverty, whose local coefficients are mostly negative while switched to positive after the start of its last quantile; terraced dwellings, whose local coefficients are mostly positive though with nearly half of the first quantile being negative; households of 3 or more people, mostly positive while nearly half of the first quantile being negative, which is similar to terraced dwellings though less influential overall; and renewable energy, which has negative local coefficients in all of its first quantile while others being positive.

For MGWR, 3 variables have spatially varied positive effects: 4-or-more bedrooms, unemployment, terraced dwellings; whilst spatially varying negative effects are found with social-rented and district heating. Spatially heterogeneous bidirectional relationships are found with 5 variables. For dwelling energy efficiency, different from all negative GWR local coefficients, the last quantile of its MGWR local coefficients is surprisingly positive. For households of 3 or more people, over half of its local coefficients are negative while the rest being positive, which is also different from GWR results with far fewer negative coefficients. For income, very different from all negative coefficients in GWR results, in MGWR most of its coefficients remain positive while nearly half of its first quantile negative. For fuel poverty, like households of 3 or more people, nearly half of its coefficients are negative while the rest positive, in GWR only some of the last quantile turned positive. For renewable energy, also different from GWR, in MGWR nearly half are positive while the rest of its local coefficients negative.

### Spatial distribution of local relationships in MGWR

To further investigate the spatial variations in the relationship between domestic energy consumption and explanatory variables discovered in MGWR, choropleth maps of standardised local coefficients are developed to examine the spatial distribution of local relationships.

**Fig. 4:**
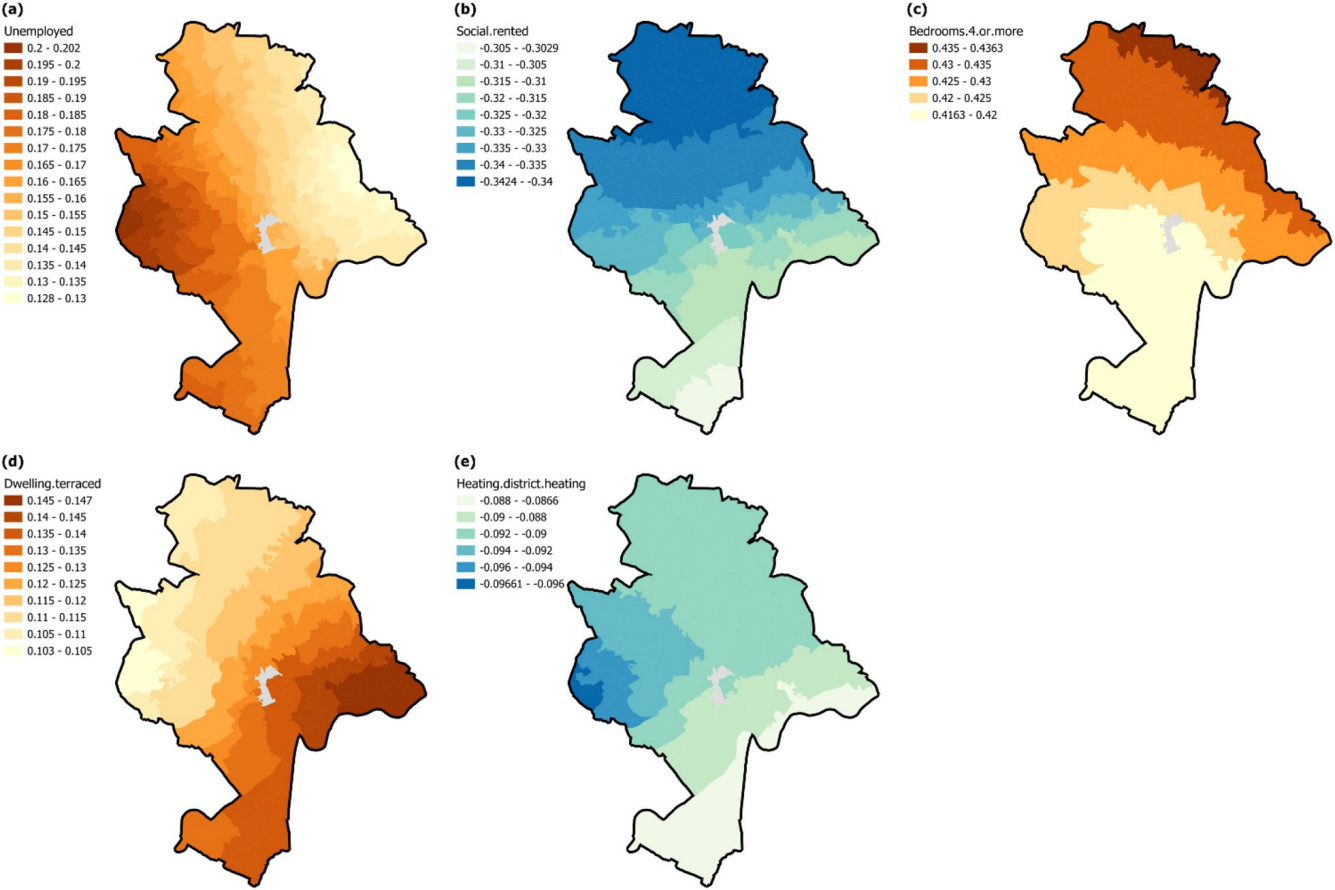
The spatially varying effects of explanatory variables on domestic energy consumption in MGWR: spatial distribution of standardised local coefficients of **a** unemployed; **b** social rented; **c** dwellings with 4 or more bedrooms; **d** terraced dwellings; **e** central heating type of district heating

Figure 4 maps standardised local coefficients of explanatory variables with spatially varying relationships across Nottingham LSOAs. A higher percentage of unemployment (Fig. 4a) elastically drives higher domestic energy consumption (standardised local coefficients from 0.128 to 0.202), with more influential effects in western Nottingham and decreasing towards eastern Nottingham. On the other hand, a larger percentage of social-rented (Fig. 4b) is elastically associated with lower domestic energy consumption (standardised local coefficients from −0.3424 to −0.3029), with more influential effects in northern Nottingham and decreasing towards southern Nottingham. The percentage of dwellings with 4 or more bedrooms (Fig. 4c) shows elastically positive effects (standardised local coefficients from 0.4163 to −0.4363), with a similar spatial pattern to social rented. The last two dwelling variables display reverse spatial patterns in their effects on domestic energy consumption, while the percentage of terraced dwellings (Fig. 4d) has elastically positive effects (standardised local coefficients from 0.103 to 0.147), with most influential in the eastern and southern then descending towards the north-western. Whereas percentage of district heating (Fig. 4e) has elastically negative effects (standardised local coefficients from −0.09661 to −0.0866), which is most influential in the western and northern areas then descending towards the south-eastern Nottingham LSOAs.

**Fig. 5:**
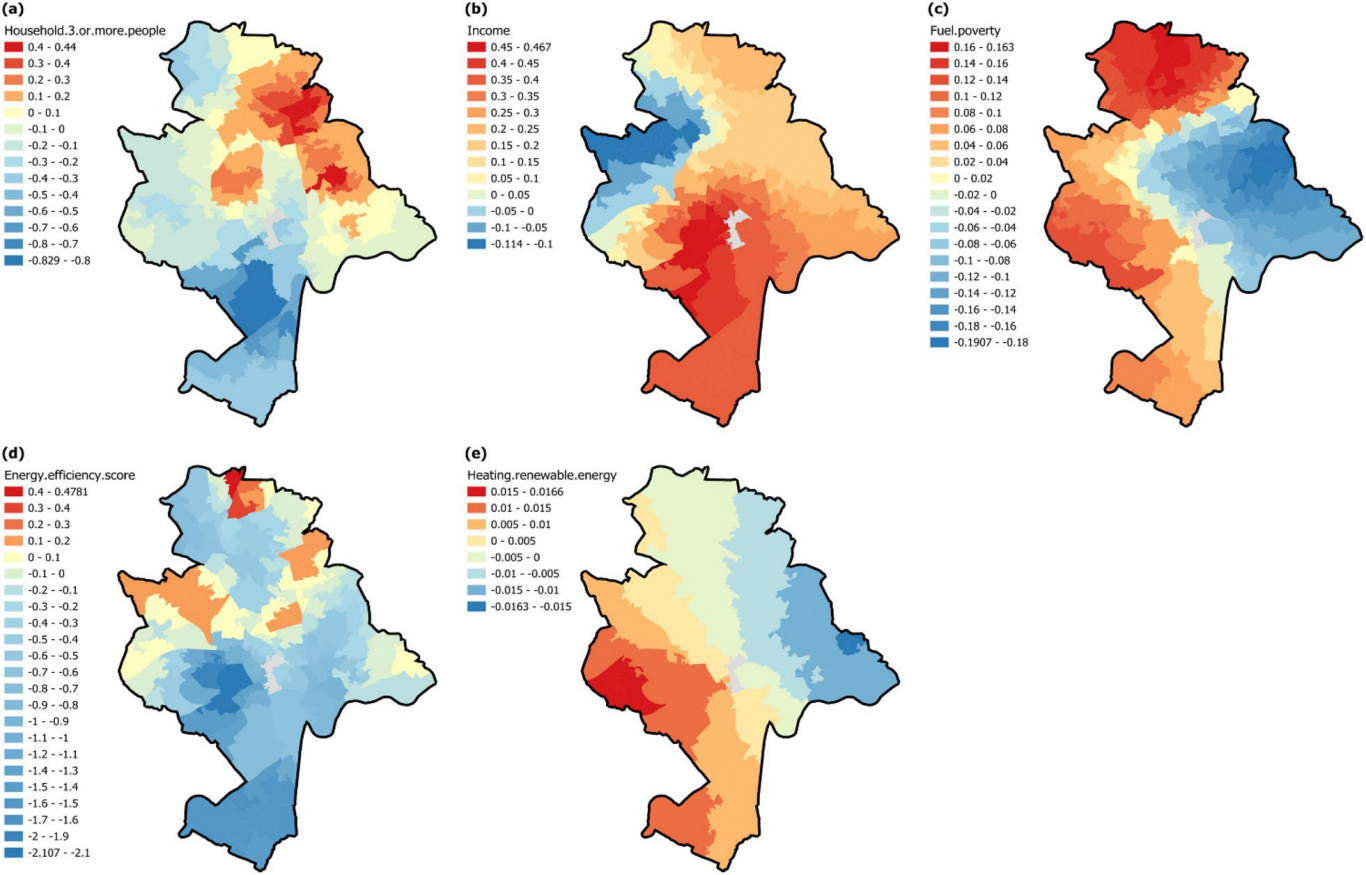
The spatially heterogeneous effects of explanatory variables on domestic energy consumption in MGWR: spatial distribution of standardised local coefficients of **a** households with 3 or more people; **b** income; **c** fuel poverty; **d** dwelling energy efficiency; **e** central heating with renewable energy

Figure 5 maps the standardised local coefficients of explanatory variables with spatially heterogeneous relationships across Nottingham LSOAs. Among them the percentage of households with 3 or more people (Fig. 5a) and dwelling energy efficiency (Fig. 5d), with standardised local coefficients from −0.829 to 0.44 and from −2.107 to 0.4781, respectively, demonstrate similar spatial patterns: a smaller proportion of LSOAs with positive effects are scattered in the north and towards the periphery, while a larger proportion of LSOAs with negative effects cover the rest. The other three variables share the spatial patterns that diverge in certain directions. Specifically, income (Fig. 5b) (standardised local coefficients from −0.114 to 0.467) positively affects domestic energy consumption in southern, eastern, and north-eastern Nottingham with decreasing influence, then turned to negative effects with increasing influence towards the north-western. Fuel poverty (Fig. 5c) and renewable energy (Fig. 5e) (standardised local coefficients from −0.1907 to 0.163 and from −0.0163 to 0.0166, respectively), have similar spatial directions of divergence: domestic energy consumption is positively affected towards southern and eastern with increasing influence while negatively so towards western LSOAs, with the difference that effects of fuel poverty are also influentially positive in northern LSOAs, though this is mostly negative for renewable energy.

## Discussion

### The spatially varying driving factors of domestic energy consumption

In summary, the global MLR model with spatial correlation in its residuals is insufficient to explain the relationship between domestic energy consumption and its driving factors. In contrast, the local models, GWR and MGWR, both outperform the MLR model, with MGWR providing the best model fit and shedding light on the spatial variations in this relationship.

On spatial variability of how domestic energy consumption is driven, the findings echo those of existing studies using GWR in two other countries. Mashhoodi et al. [84] on household energy consumption and Mashhoodi and van Timmeren [85] on respective household gas and electricity consumption, using GWR at neighbourhood level in the Netherlands, both find how consumption is driven varies spatially by environment, dwelling, and household characteristics. Similarly, Jo and Kim [83], using GWR at administrative district level in Seoul, Korea, find influences of demographic, socio-economic, building, and environmental variables on electricity consumption varies spatially. On the different spatial scales of how driving factors influence domestic energy, the findings echo those of Jo and Kim [83], whose MGWR finds household size of more than three people have smaller space scale, though differ from them on income with larger spatial scale.

This study does have several limitations. For data used, income data are from 2020, whereas all other variables are from 2021. On data level, income and energy efficiency score are converted to LSOA level from their original sources at the less granular MSOA level. For study area coverage, Nottingham is only one of the cities in England and more urban and rural areas could be studied.

The findings are generally consistent with existing research, while offering nuanced insights into aspects that have produced inconclusive results in other studies. Below, the potential reasons and lessons learned from the discovered local dynamics are further discussed, followed by proposed policy actions for fair and effective domestic energy decarbonisation and transition to net zero.

### Dealing with socio-technical uncertainties: renewable energy and district heating

For dwelling characteristics contributing to socio-technical uncertainties in domestic energy consumption, expected results are found for two variables, consistent with other studies: a higher percentage of dwellings with higher-than-average bedrooms (4 or more) leads to higher consumption [12, 16], while a higher percentage of terraced dwellings with shared walls is linked to lower consumption [15]. However, for renewable energy, this study unexpectedly finds that in almost half of Nottingham LSOAs, a higher percentage of households with renewable energy as central heating corresponds to higher consumption. This may suggest that the energy efficiency of renewable energy systems and dwellings in those areas is not adequate to realise the potential of renewable heating for decarbonisation [142], or the residents are not using the renewable heating systems correctly, possibly due to a lack of understanding of how to [143]. For these local areas, this study recommends promoting information on the efficient usage of renewable heating systems and rolling out energy efficiency schemes.

For district heating, its consistent negative spatially varied effects on domestic energy consumption across all Nottingham LSOAs are as expected. Considering domestic energy consumption in the data is calculated as the total of gas and electricity consumption, and DH supplies hot water and space heating in homes [144], effects of the DH variable represent low-carbon energy consumption for hot water and heating in places with DH [145]. DH's MGWR local coefficients indicate that a 1% increase in households with DH results in an average reduction in domestic energy consumption of 61,549.6 kWh, with spatially varied reductions ranging from 64,505.4 to 68,662.5 kWh (Table 6) across Nottingham LSOAs. This indicates district heating is very beneficial for decarbonising energy consumption. Considering the mean coverage rate of DH is less than 2.6% (Table 2) across Nottingham LSOAs, to strengthen DH's beneficial effects on decarbonisation discovered for all local areas, this study recommends extending existing Nottingham low-carbon DH networks and the development of new DH projects.

### Addressing socio-economic uncertainties: unemployment and social housing

For household characteristics contributing to socio-economic uncertainties in domestic energy consumption, unemployment is found to have consistent positive spatially varied effects, which echoes previous research findings [146]. This may result from occupancy patterns in households of different employment status, with the unemployed having the longest active hours [4]. To benefit unemployed households and others on low incomes, this study recommends promoting subsidised schemes to switch central heating systems to renewable energy and district heating but also to promote domestic energy efficiency improvements.

On social housing, this study finds a higher percentage of social rented has consistent negative spatially varied effects on domestic energy consumption, echoing existing literature [14]. This may result from the overall better energy efficiency of social housing compared to other housing tenures in England [109]. However, previous research on Nottingham social housing found that while retrofit schemes improved dwelling energy efficiency, they did not deliver the expected reduction in energy costs for social-rented households [147]. This indicates the finding on social housing may also result from potential energy needs not being adequately fulfilled due to economic restraints. To benefit social-rented households, this study suggests providing incentives to reduce energy bills in combination with energy efficiency improvement schemes.

### Recognising dynamics across socio-economic and socio-technical uncertainties: income, dwelling energy efficiency, and fuel poverty

The dynamics across household and dwelling factors contributing to socio-economic and socio-technical uncertainties in driving domestic energy consumption [9] are further highlighted in the findings. For instance, a higher-than-average household size (3 or more people) is found to have a spatially heterogeneous bidirectional relationship with domestic energy consumption. This finding provides a nuanced interpretation of the currently inconclusive results in existing literature [4, 12, 13, 18, 19]. It also reflects how household size affects domestic energy consumption, which is influenced by the dynamics between household size and other factors such as dwelling types and the number of rooms [146]. Similar dynamics are observed in income, dwelling energy efficiency, and fuel poverty.

Income is identified as a significant factor positively driving domestic energy consumption in existing studies [4, 12, 15, 16], and this study’s findings are consistent with that finding for most Nottingham LSOAs. However, for the remaining LSOAs, income exhibits spatially varied negative relationships, similar to the pattern observed with dwelling energy efficiency. Consistent with previous studies [14, 15, 18], this study finds that better energy efficiency generally leads to lower domestic energy consumption in most Nottingham LSOAs. However, in a quarter of LSOAs, the local patterns are reversed, with better dwelling energy efficiency associated with higher domestic energy consumption. These irregularities reflect the complex dynamics across household and dwelling factors that contribute to socio-economic and socio-technical uncertainties in driving domestic energy consumption. Webber et al. [148] find that the actual impacts of energy efficiency schemes on domestic energy consumption vary by income, with low-income areas showing consistent impacts, while areas with middle and higher incomes experience higher impacts. Bergman and Eyre [149] discuss the situation of socio-technical ‘lock-in’, where the industry’s focus on maximising energy sales and individuals’ lack of access to the best information on energy saving contributes to the disconnect between occupant behaviour and domestic energy consumption in the UK. Pelenur and Cruickshank [150] explore the residential energy efficiency gap, finding a strong association between demographic variables and barriers to installing energy efficiency measures in dwellings cross large UK cities.

Considering fuel poverty is the situation where access to adequate energy is lacking, and the indicator LILEE estimates fuel poverty by low income and low energy efficiency [116], this study expected higher percentage of fuel poor households leads to lower consumption, as they cannot afford to use as much energy as they need. This is found to be the case for only half of Nottingham LSOAs in eastern Nottingham. For the rest, however, a higher percentage of fuel poor households spatially leads to higher energy consumption. This may suggest while fuel poor households cannot afford to use adequate energy, they still must use more to keep at even an inadequate level despite the rising energy costs [151]. This may indicate the strong influence of inefficient housing energy efficiency in these areas, and further reflects the importance to recognise the needs of vulnerable groups, and provide them with access to energy efficiency schemes, so that co-benefits of improved health and reduced emissions can be generated [152].

Therefore, for local areas where better energy efficiency leads to higher energy consumption, and places where lower income leads to higher consumption, this study recommends promoting information on energy saving measures and making them accessible for people to benefit from, while rolling out subsidised programmes to switch central heating to renewable energy and district heating. For local areas where a higher level of fuel poverty leads to a higher energy consumption, this study recommends prioritising domestic energy efficiency improvement with incentives for energy cost reduction.

## Conclusions

This study develops local spatial statistical models GWR and MGWR to investigate spatial variations in the relationship between domestic energy consumption and its driving factors on household and dwelling characteristics across Nottingham LSOAs. As one the first UK studies using MGWR on domestic energy consumption, this research addresses gaps in spatial design and local variability identified in reviewed literature. Results from the best model MGWR show widespread spatial variability in all variables tested for this relationship, and counterintuitively, spatial heterogeneity in half of the tested explanatory variables.

Whilst this study focuses on Nottingham, the findings may also have significance for policy and future research on domestic energy consumption covering varied areas and levels of analysis. Notably, this study finds domestic energy consumption is driven by factors at different spatial scales, including some very locally, and with patterns that are spatially varied or even spatially heterogeneous for characteristics on both dwellings and households. This suggest the “one-size-fit-all” type of policy plans may not be the most suitable option for achieving envisioned outcomes of rapid and fair domestic energy decarbonisation. Based on the findings, this study therefore recommends placed-based approaches and more local deliberations in devising policies for domestic energy decarbonisation and more broadly energy transition to net zero. Specifically, this study makes the following place-based policy suggestions to address socio-technical and socio-economic uncertainties for effective and fair domestic energy decarbonisation in Nottingham. Where district heating is in place, it should be extended. The effects of renewable energy sources are ambiguous across different groups: where energy costs increase, households need focused support and incentives to get bills down. Alongside focusing on renewable energy and district heating, it is important not to neglect energy efficiency upgrades to properties, especially for those on low incomes.

This study highlights potential avenues for future research. Its approach could be expanded to wider study areas, such as including a collection of local authorities in both urban and rural areas, to make comparisons across different places and to help inform a wider range of stakeholders. The approach may also be applied to other spatial levels to further investigate local relationships and how they vary spatially. For instance, this could involve examining more granular levels such as Output Areas or postcodes. Future research areas include examining final energy demand issues related to conversion losses and heating types, analysing the dwelling stock structure in domestic energy consumption in greater details, and investigating DH energy usage at local and household levels to understand its impact on energy decarbonisation.

## Data Availability

This study uses publicly available data sources as outlined in section ‘Data Collection’. The data on Nottingham analysed in this study can be accessed via http://doi.org/10.17036/researchdata.aston.ac.uk.00000629.
